# Metabolic Syndrome Among Testicular Cancer Survivors: Long‐Term Follow‐Up of the Veterans Affairs Health System

**DOI:** 10.1002/cam4.70858

**Published:** 2025-04-21

**Authors:** Dhruv Puri, Paul Riviere, Margaret Meagher, Kylie Morgan, Tyler Nelson, Kit Yuen, Kshitij Pandit, Nuphat Yodkhunnatham, Jacob Taylor, Daniel Herchenhorn, Tyler Stewart, Juan Javier‐Desloges, Amirali Salmasi, Rana R. McKay, Sean Q. Kern, Heather Hofflich, Frederick Millard, Brent Rose, Aditya Bagrodia

**Affiliations:** ^1^ Department of Urology UC San Diego School of Medicine La Jolla California USA; ^2^ Department of Radiation Medicine and Applied Sciences UC San Diego School of Medicine La Jolla California USA; ^3^ VA Hospital San Diego San Diego California USA; ^4^ Department of Urology UTSW School of Medicine Dallas Texas USA; ^5^ Division of Hematology and Oncology, Department of Medicine UC San Diego School of Medicine La Jolla California USA; ^6^ Department of Urology Uniformed Services University and Walter Reed National Military Medical Center Bethesda Maryland USA; ^7^ Division of Endocrinology, Department of Medicine UC San Diego School of Medicine La Jolla California USA

**Keywords:** chemotherapy, metabolic syndrome, oncology survivorship, testicular cancer survivors

## Abstract

**Background:**

The 5‐year survival rate for patients with testicular germ cell tumors (TC) is excellent. However, these survivors are at an increased risk for metabolic syndrome (MetS), a significant source of morbidity and precursor to cardiovascular disease. This study investigates the incidence of MetS in TC survivors compared to matched controls.

**Methods:**

A retrospective analysis was conducted using the Veterans Affairs national database. The incidence of MetS was compared between 2021 TC survivors and 7595 matched controls. MetS was identified via diagnostic codes and medication use, requiring at least three of five criteria: insulin resistance, dyslipidemia, central obesity, and hypertension. Statistical analysis included chi‐squared and *t*‐tests for demographic comparisons, and Cox regression for outcome associations.

**Results:**

TC survivors exhibited a greater prevalence of MetS components than controls, specifically hyperglycemia (28.4%), low HDL levels (59.8%), hypertriglyceridemia (8.0%), and abdominal obesity (27.3%), except for hypertension. Over 5 and 10 years, the cumulative incidence of MetS in TC survivors was 17.0% and 27.8%, compared to 1.9% and 2.8% in controls. Multivariate regression showed an increased incidence of MetS in TC survivors (HR = 19.02, 95% confidence interval [CI]: 16.31–22.19, *p* < 0.001). Chemotherapy (HR = 1.28, 95% CI: 1.04–1.57, *p* = 0.017) and increasing age (HR = 1.04, 95% CI: 1.04–1.06, *p* < 0.001) were associated with a higher risk.

**Conclusions:**

TC survivors have a substantial risk of MetS with a higher occurrence of most MetS components, barring hypertension. Comprehensive metabolic health monitoring is crucial in TC survivorship care. Integrating vigilant screening and preventive strategies can mitigate MetS development in this population.

## Graphical abstract

Testicular cancer survivors face a significantly higher risk of developing metabolic syndrome compared to non‐cancer controls. Vigilant metabolic health monitoring and preventive strategies are crucial in survivorship care to mitigate long‐term cardiovascular risks.

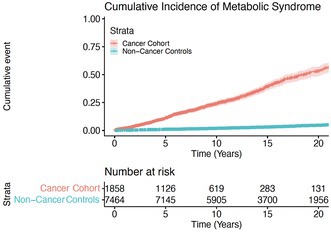

## 1 Introduction

Testicular cancer (TC) of germ cell origin, most commonly diagnosed in men under 40, now has a 5‐year survival rate exceeding 95% due to the integration of effective cisplatin‐based chemotherapy protocols and surgery [1, 2, 3, 4, 5]. Despite this, survivors face a heightened susceptibility to enduring adverse effects of chemotherapy that can arise long after treatment completion [6]. Metabolic syndrome (MetS) has been increasingly recognized as a long‐term consequence of bleomycin‐ and cisplatin‐containing chemotherapy in TC [7]. MetS is a clustering of risk factors for cardiovascular disease that includes increased blood pressure, high blood sugar, excess body fat around the waist, and abnormal cholesterol or triglyceride levels [8]. While the association between MetS and various cancers has been increasingly recognized, its prevalence and impact in TC patients still require further clarification [9, 10]. TC survivors are also at an elevated risk of cardiovascular morbidity and mortality, which significantly impacts their long‐term health outcomes and is a cause of late mortality in cancer survivors [11]. This study aims to investigate the incidence of MetS and its components in a large national cohort of TC survivors compared to a matched non‐cancer control group.

## 2 Materials (Patients) and Methods

### 2.1 Data Source

In this retrospective analysis, data were obtained from the Veterans Affairs (VA) national electronic health record system, specifically the Corporate Data Warehouse, and the VA Central Cancer Registry, accessed via the VA Informatics and Computing Infrastructure (VINCI) [12]. Due to the retrospective design of the study and the minimal risk posed to participants, informed consent was not required. The VA Central Cancer Registry adheres to the standards established by the North American Association of Central Cancer Registries for the identification and reporting of incident cancer cases and treatment modalities. Ethical approval for this investigation was obtained from the San Diego VA Institutional Review Board. Our cohort has been previously described [13].

Follow‐up in the VA healthcare system is guided by standardized clinical practice guidelines, which include regular screening for cardiovascular risk factors such as hypertension, hyperlipidemia, and diabetes [14]. Screening intervals are determined by individual patient risk profiles and comorbidities, ensuring that high‐risk individuals receive more frequent monitoring. Cardiovascular risk factors are identified using ICD diagnostic codes and prescription data, reflecting real‐world clinical management practices. These practices are supported by the VA's robust clinical guidelines for chronic disease management, ensuring uniformity in the identification and treatment of cardiovascular conditions across the system.

### 2.2 Study Population

In total, 2824 patients diagnosed with TC were identified using ICD‐10 codes. The exclusion criteria were non‐germ cell tumor histology (*n* = 247), incomplete staging information (*n* = 527), and incomplete follow‐up data (*n* = 29), resulting in a final cohort of 2021 patients. These individuals were age‐ and race‐matched to a control group of 7595 men without a history of TC. Demographic data, including race (white, other, Native American, African American, Asian Pacific Islander), ethnicity (Hispanic, non‐Hispanic), marital status (single, married, divorced/separated/widowed), history of tobacco use (current, former, never, unknown), and employment status (employed, unemployed, retired), were extracted from the national health record. Information regarding cancer, such as the year of diagnosis, age of the patient at diagnosis, clinical T staging (T1, T2, T3, T4), clinical N staging (N0, N1, N2, N3), clinical M staging (M0, M1), and treatment modalities, were obtained from the VA cancer registry.

### 2.3 Outcomes

The primary endpoint of this study was the development of MetS, established by the National Cholesterol Education Program Adult Treatment Panel III (NCEP‐ATP III) as the presence of three or more of the following criteria: insulin resistance, dyslipidemia, central obesity, and hypertension [8, 15]. To operationalize the criteria for MetS, we utilized International Classification of Diseases (ICD) codes to identify cases of insulin resistance, dyslipidemia, central obesity, and hypertension. The presence of these conditions was inferred from the ICD diagnosis codes. Additionally, we considered the prescription and administration of specific medications known to treat the aforementioned conditions for a duration of at least 6 months as a proxy for the presence of each component. This methodology has been previously described [16]. International Statistical Classification of Diseases Codes from editions 9 and 10 are provided in Table S1, and medications used to treat these diagnoses are listed in Table S2 [17]. Patients with a prior diagnosis of MetS were excluded from the analysis to focus on cases developing after TC diagnosis, ensuring alignment with a de novo assessment methodology. Time‐to‐event was calculated from the date of diagnosis to the occurrence of the third criterion of MetS or censoring at the time of the last follow‐up. For the non‐cancer control cohort, an index date was created that corresponded to the matched TC patient's date of diagnosis, and time‐to‐event was defined as the index date until the date of development of MetS or last follow‐up. Outcomes were reported as cumulative incidences.

### 2.4 Statistical Analysis

To ascertain differences in categorical variables between cancer patients and non‐cancer controls, the chi‐squared test was utilized. Continuous variables were assessed using the independent samples *t*‐test. The impact of treatment regimens on outcomes was evaluated through Cox proportional hazards models, incorporating clustering for matched analysis with non‐cancer controls. We conducted an analysis of all TC patients based on the receipt of chemotherapy. Estimated hazard ratios (HRs) and their corresponding 95% confidence intervals (CIs) were reported. Cumulative incidences were calculated to establish rates of primary and secondary outcomes and for data visualization. All statistical tests were two‐sided, with a significance threshold set at *p* < 0.05. The analysis was performed using R Studio version 3.5.1 (The R Foundation, Vienna, Austria).

## 3 Results

In total, 2021 patients with TC and 7595 non‐cancer control patients were analyzed. The median age at diagnosis was 40.3 years (IQR: 31.3–51.7). The median follow‐up duration for the TC cohort was 7.91 years (IQR: 3.51–13.37 years, range: 0.5–25.3 years) from the time of diagnosis. Table 1 displays demographic and clinical information for the cancer cohort. Most of the cancer cohort, 1814 patients (89.8%), were white, with 125 patients (6.2%) identifying as Hispanic. 73.4% of patients had a clinical stage I, 13.6% had clinical stage II, and 12.9% had clinical stage III at diagnosis. 840 (41.6%) of patients underwent surgery (orchiectomy) alone, 471 (23.3%) underwent surgery and radiation, 624 (30.9%) underwent surgery and chemotherapy, and 86 (4.3%) of patients underwent surgery, chemotherapy, and radiotherapy. In total, 1339 patients (66.3%) had primary seminoma histology.

**TABLE 1:** Demographic and clinical characteristics for the cohort of testicular cancer survivors.

Variable	*N* (%)
Total number of patients	2021
Age at diagnosis, meDIAN (IQR)	40.3 (31.3–51.7)
Year of diagnosis	
1990–1999	403 (19.9)
2000–2009	698 (34.5)
2010–2021	920 (45.5)
Race	
Asian and Pacific Islander	25 (1.2)
Black	120 (5.9)
Native American	14 (0.7)
Other	48 (2.4)
White	1814 (89.8)
Hispanic	125 (6.2)
Martial status	
Divorced/separated/widowed	599 (29.6)
Married	779 (38.5)
Not married	643 (31.8)
Employment status	
Employed	745 (36.9)
Not employed	926 (45.8)
Retired	238 (11.8)
Unknown	112 (5.5)
Smoking history	
Current	808 (40.0)
Never	586 (29.0)
Prior	337 (16.7)
Unknown	290 (14.3)
Seminoma (%)	1339 (66.3)
Clinical stage	
1	1485 (73.5)
2	275 (13.6)
3	261 (12.9)
T stage	
T0	18 (0.9)
T1	1257 (62.2)
T2	397 (19.6)
T3	89 (4.4)
T4	14 (0.7)
Missing	246 (12.2)
N stage	
N0	1406 (69.6)
N1	145 (7.2)
N2	143 (7.1)
N3	100 (4.9)
Missing	227 (11.2)
M stage	
M0	1631 (80.7)
M1	197 (9.7)
Missing	193 (9.5)
Treatment	
Surgery alone	840 (41.6)
Surgery and chemotherapy	624 (30.9)
Surgery and radiation	471 (23.3)
Other^a^	86 (4.3)

^a^ All other combinations of chemotherapy, radiation, and surgery.

Table 2 shows the frequency of the determinants of MetS between the cancer and non‐cancer cohorts. The frequency of high blood glucose (28.4 versus 7.3%), low HDL (59.8 versus 7.0%), hypertriglyceridemia (8.0 versus 0.8%), and abdominal obesity (27.3 versus 3.4%) was all significantly higher in the cancer cohort than in the non‐cancer cohort, respectively (*p* < 0.001 for all). Hypertension was not significantly different between the two cohorts (Cancer 57.8 versus Non‐cancer 58.9%, *p* = 0.363). In total, 503 TC patients (24.9%) developed MetS.

**TABLE 2:** Prevalence of the components of metabolic syndrome in the cancer and non‐cancer cohort.

	Non‐cancer *N* (%)	Cancer *N* (%)	*p*
Total number	7595	2021	
High fasting glucose	552 (7.3)	573 (28.4)	< 0.001
High blood pressure	4477 (58.9)	1168 (57.8)	0.363
Low HDL‐C	534 (7.0)	1209 (59.8)	< 0.001
Hypertriglyceridemia	57 (0.8)	162 (8.0)	< 0.001
Abdominal obesity	256 (3.4)	552 (27.3)	< 0.001

*Note*: Cut‐off values: Hypertension (≥ 130/85 mmHg), Elevated fasting glucose (≥ 100 mg/dL), Low HDL cholesterol (< 40 mg/dL HDL‐C), Hypertriglyceridemia (≥ 150 mg/dL), Abdominal obesity (waist circumference ≥ 102 cm).

**FIGURE 1:**
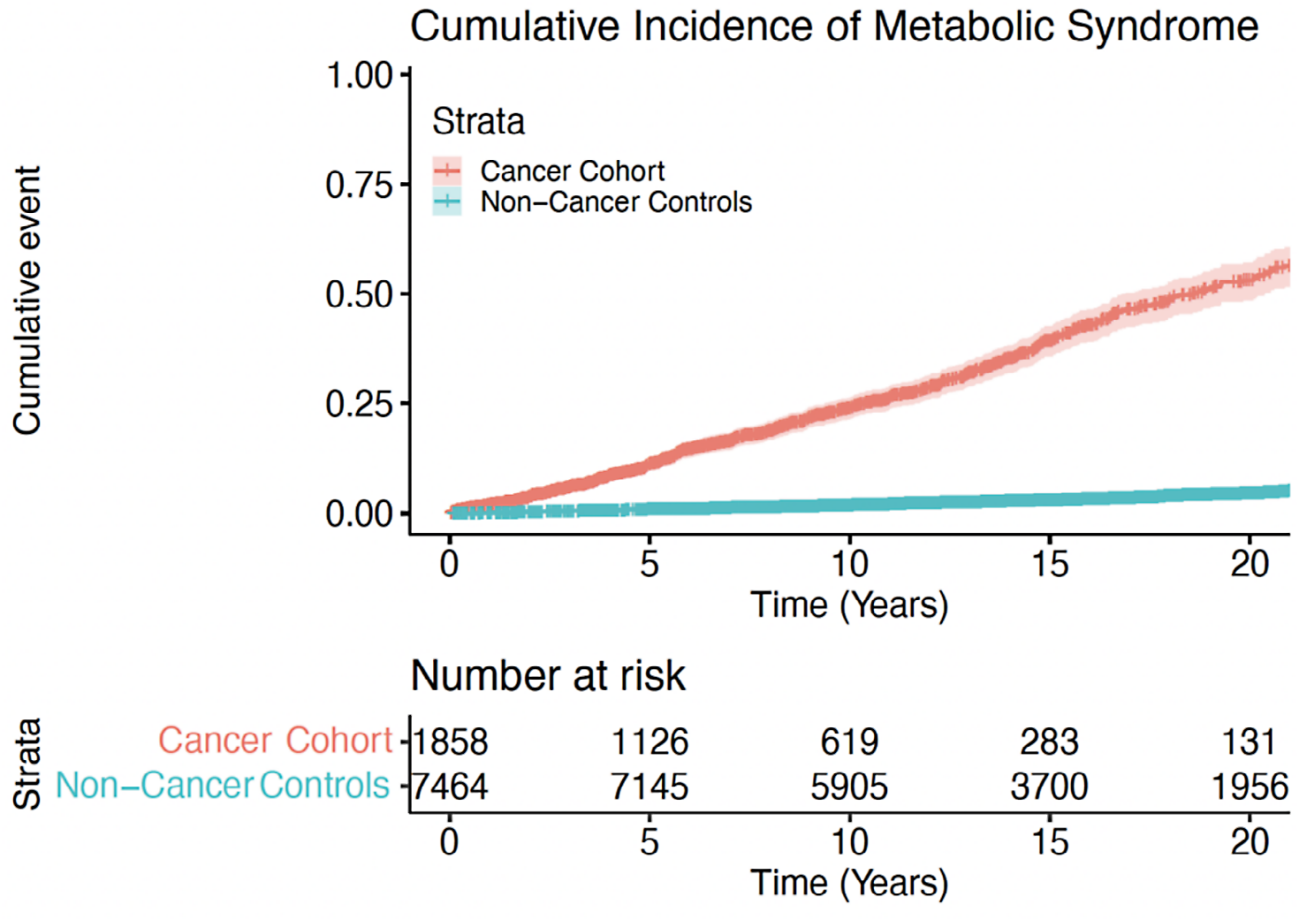
Cumulative incidence of metabolic syndrome stratified by testicular cancer and healthy patients.

On clustered univariable cox regression analysis, cancer diagnosis (HR = 16.65, *p* < 0.001) was associated with increased cumulative incidence of *de novo* MetS (Table 3). In multivariable regression adjusting for age, race, and year of diagnosis, cancer survivors had a HR of 19.02 (16.31–22.19, *p* < 0.001). Additionally, increasing age (HR: 1.04, 95% CI: 1.04–1.05, *p* < 0.001) and year of diagnosis (years 2000–2009, HR: 1.49, 95% CI: 1.24–1.78, *p* < 0.001; years 2010–2021, HR: 1.38; 95% CI: 1.09–1.74, *p* = 0.008) were also associated with increased cumulative incidence of de novo MetS (Table 3). The 5‐year cumulative incidence of de novo MetS was 17.0% for cancer patients and 1.9% for non‐cancer patients (*p* < 0.001); 10‐year cumulative incidence of de novo MetS was 27.8% for cancer patients and 2.8% for non‐cancer patients (*p* < 0.001) (Figure 1). Cox regression analysis demonstrated receipt of chemotherapy (HR = 1.28, 95% CI: 1.04–1.57, *p* = 0.017) and increasing age (HR = 1.04, 95% CI: 1.04–1.06, *p* < 0.001) to be associated with increased risk of development of MetS (Table 4).

**TABLE 3:** Univariable and multivariable analysis of risk factors for the development of metabolic syndrome in testicular cancer survivors and non‐cancer controls.

	HR (95% CI)	*p*
*Univariable analysis*		
Cancer cohort (ref: non‐cancer)	16.65 (14.31–19.36)	< 0.001
*Multivariable analysis*		
Cancer cohort (ref: non‐cancer)	19.02 (16.31–22.19)	< 0.001
Race (ref: Asian and Pacific Islander)		
Black	1.09 (0.44–2.72)	0.851
Native American	1.39 (0.33–5.82)	0.652
Other	1.11 (0.42–2.92)	0.832
White	0.87 (0.36–2.1)	0.759
Age at diagnosis (ref: <= 30)		
31–40	2.65 (1.93–3.66)	< 0.001
41–50	4.09 (2.98–5.60)	< 0.001
> 50	6.68 (4.91–9.09)	< 0.001
Year of diagnosis (ref: 1990–1999)		
2000–2009	1.49 (1.24–1.78)	< 0.001
2010–2021	1.38 (1.09–1.74)	0.008

**TABLE 4:** Effects of chemotherapy on risk of metabolic syndrome.

	HR	95% CI	*p*
Received chemotherapy (ref: no chemotherapy)	1.28	1.04–1.57	0.0169
Age at diagnosis	1.05	1.04–1.06	< 0.001
Clinical stage 2 (ref: clinical stage 1)	1.05	0.83–1.34	0.6717
Clinical stage 3	0.86	0.64–1.17	0.3366

## 4 Discussion

Our study is the largest to date quantifying the incidence of *de novo* MetS in TC survivors, highlighting the significantly elevated risk as compared to matched non‐cancer controls. The development of three of the components of MetS was significantly higher in the cancer cohort compared to the non‐cancer cohort: namely elevated rates of high blood glucose, low HDL, hypertriglyceridemia, and abdominal obesity. Hypertension rates were similar between the two cohorts. Cox regression analysis revealed that a cancer diagnosis was clinically and statistically significantly associated with an increased cumulative incidence of de novo MetS. This increased risk of developing MetS after the diagnosis of TC appeared to be in part associated with receipt of chemotherapy as a part of TC‐directed therapy.

In the past two decades, there has been an increased concern about the risk of cardiovascular comorbidities in TC survivors, especially the development of MetS, which can increase the risk of the development of cardiovascular disease, diabetes, second primary malignancies, and all‐cause mortality [18, 19, 20]. In a single center analysis at University Medical Center Groningen, de Haas analyzed a cohort of 173 TC survivors against 1085 controls and found that the TC survivors had an increased risk of developing MetS (OR = 2.2, 95% CI: 1.5–3.3) [21]. This was built upon by researchers from Leiden University Medical Center where Willemse et al. demonstrated a similar trend in a cohort of 255 testicular germ cell tumor survivors (OR = 1.9; 95% CI: 1.1–3.2) compared to controls [22]. Our own analysis expands on this with a larger national cohort of 2021 TC patients compared with 7595 controls with an HR of 19.02 (95% CI: 16.3–22.2; *p* < 0.001).

The literature on the diagnostic components of MetS in TC survivors is controversial, with our study noting increased rates for most determinants but not for high blood pressure. de Haas et al. found that TC survivors had a higher prevalence of: high blood pressure 59% (*p* < 0.0001), low HDL cholesterol 44% (*p* = 0.003), central obesity 17% (*p* = 0.005), and high glucose 14% (*p* = 0.002) but not high triglycerides 29% (*p* = 0.424) [21]. Frequencies in the control cohort were not reported. Alternatively, Zaid et al. examined 486 TC survivors and found that patients had higher rates of hypertension (43.2% vs. 30.7%; *p* < 0.001), elevated total cholesterol levels (26.3% vs. 11.1%; *p* < 0.001), and body mass index ≥ 25 kg/m^2^ (75.1% vs. 69.1%; *p* = 0.04). However, TC patients were less likely to have decreased HDL levels (23.7% vs. 34.8%; *p* < 0.001) or abdominal obesity (28.2% vs. 40.1%; *p* < 0.001) [23]. Our results showed higher rates of each determinant in the TC cohort, except for high blood pressure, which was relatively common in both groups.

The etiology of MetS in TC patients has been postulated to be the hormonal sequalae of TC or the receipt of platinum‐based chemotherapy regimens. Bogefors et al. examined 92 TC patients and found that hypogonadism (total testosterone < 10 nmol/L and/or LH > 10 IU/L and/or androgen replacement) also presented with increased risk (OR = 4.4; *p* = 0.01) of MetS [24]. Alternatively, in a national follow‐up study, Haugnes et al. examined 1463 TC survivors and found that patients receiving cisplatin > 850 mg had a higher odds of developing MetS (OR = 2.6; 95% CI: 1.1–6.0) compared to surgery, radiotherapy, and cisplatin ≤ 850 mg [25]. Our own results mirrored their findings, with receipt of chemotherapy having a higher risk of developing MetS (HR = 1.3, 95% CI: 1.0–1.6, *p* = 0.017). Willemse et al. remains the only study to examine both chemotherapy and low testosterone in the context of MetS in TC survivors and found that there were no differences in gonadal hormone levels between patients treated with adjuvant single‐dose carboplatin compared to other modalities [22]. It has been shown that cisplatin can be detectable in the plasma up to 20 years post chemotherapy in TC patients, and hypogonadism is a common cancer‐related effect to TC [26, 27, 28, 29]. Further work is required to clarify the true etiology and elaborate on this finding.

The patient population is a strength of this study because of the VINCI dataset's origins from the VA's comprehensive, nationwide health system. This system's wide reach allows us to gather a substantial and diverse cohort. Historical analyses using VINCI have consistently delivered robust, externally valid results [30, 31, 32]. Furthermore, the VA's robust clinical guidelines for chronic disease management, particularly regarding MetS components like diabetes, dyslipidemia, hypertension, and obesity, result in a cohort with uniform and thorough follow‐up and disease management [14]. This translates into a cohort well managed for chronic conditions, which is beneficial for conducting comprehensive screenings for cardiovascular disease within the MetS framework.

The data underscores the importance of proactive health management in TC survivors, particularly stressing the essence of close medical follow‐up even in stage I patients treated solely with orchiectomy, as the procedure may increase the risk of hypogonadism, which is a known contributor to adverse metabolic outcomes [33]. There is a clear need for increased vigilance in screening for MetS and its components early in survivorship, given the enhanced risk. Such measures would not only aim at early detection but also guide interventions to prevent the progression of metabolic disturbances. Integrating comprehensive survivorship care with a focus on metabolic health can substantially improve long‐term outcomes and quality of life, leveraging the interdisciplinary expertise from oncology, endocrinology, cardiology, and primary care to optimize patient care post‐cancer treatment.

Our study is subject to several limitations that warrant consideration. The retrospective nature of our analysis precludes the establishment of causality between treatment for TC and the development of MetS. Furthermore, our reliance on a claims‐defined approach for identifying MetS may not accurately capture all cases, as this method depends on the presence of diagnostic codes or medication prescriptions, which might not be comprehensively recorded for all patients. The control group's lower prevalence of MetS compared to the general population may reflect selection bias or differences in healthcare utilization, potentially limiting generalizability. Additionally, the study's design did not account for longitudinal changes in the components of MetS or their treatment, nor did it include complete laboratory values for all patients, which could lead to misclassification. There is also a concern for ascertainment bias, given that patients with a history of cancer often undergo more frequent medical monitoring, potentially leading to a higher reported prevalence of MetS in this group. However, the similar rates of hypertension observed in both the cancer and control cohorts suggest comparable levels of health care engagement, which partially allays this concern. Additionally, the high absolute rate of MetS observed in the TC survivor group (which is expected to have excellent oncologic survival) should prompt close surveillance during survivorship care regardless of whether relative risk compared to non‐cancer controls is affected by ascertainment bias. Other limitations include potential unmeasured confounders such as lifestyle factors and genetic predispositions. Lastly, the follow‐up duration may be inadequate to fully capture the long‐term risks of MetS development, particularly in younger cancer survivors. Future research should aim to address these limitations through prospective studies, a more diverse patient population, and a comprehensive capture of longitudinal treatment and metabolic data.

## 5 Conclusion

This study substantiates the increased incidence of MetS among TC cancer survivors, providing the largest study to date in this population. Our results, consistent with the current body of research, suggest that metabolic health should be a key component of post‐treatment care. Implementing rigorous screening protocols and interventions for MetS in survivors is imperative to mitigate this risk. The long‐term effects of chemotherapy regimens, specifically those that include cisplatin, are noteworthy and warrant careful consideration in survivorship planning. Overall, our analysis underscores the need for a strategic approach in managing the health of testicular cancer survivors, integrating oncologic vigilance with proactive measures to combat MetS.

## Author Contributions

**Dhruv Puri:** conceptualization (equal), data curation (equal), formal analysis (equal), investigation (equal), methodology (equal), project administration (equal), resources (equal), software (equal), validation (equal), visualization (equal), writing – original draft (equal), writing – review and editing (equal). **Paul Riviere:** conceptualization (equal), data curation (equal), formal analysis (equal), methodology (equal), validation (equal), visualization (equal), writing – original draft (equal), writing – review and editing (equal). **Margaret Meagher:** conceptualization (equal), data curation (equal), formal analysis (equal), investigation (equal), methodology (equal), supervision (equal), validation (equal), writing – review and editing (equal). **Kylie Morgan:** conceptualization (equal), data curation (equal), formal analysis (equal), funding acquisition (equal), investigation (equal), methodology (equal), project administration (equal), writing – review and editing (equal). **Tyler Nelson:** conceptualization (equal), data curation (equal), formal analysis (equal), methodology (equal), project administration (equal), resources (equal), software (equal). **Kit Yuen:** conceptualization (equal), investigation (equal), methodology (equal), project administration (equal), validation (equal), writing – original draft (equal), writing – review and editing (equal). **Kshitij Pandit:** conceptualization (equal), data curation (equal), investigation (equal), methodology (equal), project administration (equal), resources (equal), writing – original draft (equal), writing – review and editing (equal). **Nuphat Yodkhunnatham:** conceptualization (equal), data curation (equal), investigation (equal), project administration (equal), writing – review and editing (equal). **Jacob Taylor:** conceptualization (equal), investigation (equal), supervision (equal), validation (equal), writing – review and editing (equal). **Daniel Herchenhorn:** conceptualization (equal), funding acquisition (equal), investigation (equal), methodology (equal), supervision (equal), validation (equal), writing – review and editing (equal). **Tyler Stewart:** conceptualization (equal), formal analysis (equal), investigation (equal), supervision (equal), validation (equal), writing – review and editing (equal). **Juan Javier‐Desloges:** funding acquisition (equal), investigation (equal), supervision (equal), validation (equal), writing – review and editing (equal). **Amirali Salmasi:** conceptualization (equal), funding acquisition (equal), investigation (equal), project administration (equal), supervision (equal), writing – review and editing (equal). **Rana R. McKay:** conceptualization (equal), funding acquisition (equal), investigation (equal), supervision (equal), validation (equal), writing – review and editing (equal). **Sean Q. Kern:** funding acquisition (equal), methodology (equal), project administration (equal), resources (equal), supervision (equal), writing – review and editing (equal). **Heather Hofflich:** conceptualization (equal), data curation (equal), investigation (equal), supervision (equal). **Frederick Millard:** conceptualization (equal), funding acquisition (equal), investigation (equal), supervision (equal), validation (equal), writing – review and editing (equal). **Brent Rose:** conceptualization (equal), data curation (equal), funding acquisition (equal), investigation (equal), methodology (equal), project administration (equal), resources (equal), supervision (equal), writing – review and editing (equal). **Aditya Bagrodia:** conceptualization (lead), funding acquisition (equal), investigation (equal), methodology (equal), project administration (lead), resources (equal), supervision (lead), validation (equal), visualization (equal), writing – original draft (equal), writing – review and editing (equal).

## Conflicts of Interest

The authors declare no conflicts of interest.

## Supporting information


Data S1.

## Data Availability

The data that support the findings of this study are available from the VA Informatics and Computing Infrastructure (VINCI) database, managed by the Department of Veterans Affairs (VA). Restrictions apply to the availability of these data, which were used under license for this study. Due to the sensitive and confidential nature of the information contained within VINCI, access is restricted to authorized individuals with the appropriate government clearance. Data are available from the authors with the permission of the Department of Veterans Affairs. Interested researchers must obtain the necessary approvals from the VA to access the VINCI database and related materials [https://www.research.va.gov/programs/vinci/].
