# Supplementary material for: Metabolic Syndrome Among Testicular Cancer Survivors: Long‐Term Follow‐Up of the Veterans Affairs Health System
Source: Cancer Med. 2025 Apr 21;14(8):e70858. doi: 10.1002/cam4.70858 (PMC12010195; doi:10.1002/cam4.70858)
Supplement: Supplementary file 1 — Data S1. [file CAM4-14-e70858-s001.docx]

Supplementary Table 1

| Risk Factor Definitions |  |  |
| --- | --- | --- |
| Risk Factor | Claims based definition | Laboratory based definition |
| Obesity | A diagnosis of unspecified or morbid obesity (ICD-9: 278.00, 278.01, ICD-10: E66) | Body Mass Index (BMI) ≥ 30 kg/m² or Waist Circumference > 40 inches (102 cm) in men or Waist Circumference ≥ 35 inches (88 cm) in women |
| Elevated fasting glucose | A diagnosis of type 2 diabetes mellitus or impaired fasting glucose (ICD-9: 250.00, 250.02, 790.21, ICD-10: E11- E1) | Fasting Glucose ≥110 mg/dL (5.6 mmol/L) |
| Elevated blood pressure | A diagnosis of hypertensive disease (ICD-9: 401.xx-405.xx, ICD 10: I10) | Blood Pressure ≥ 130/85 mm Hg |
| Low HDL-C | A diagnosis of lipoprotein deficiency (ICD-9: 272.5x, ICD-10: E78.4) | High Density Lipoprotein-Cholesterol (HDL-C) < 40 mg/dL (1.03 mmol/L) in men or HDL-C < 50 mg/dL (1.29 mmol/L) in women |
| Elevated triglycerides | A diagnosis of pure hyperglyceridemia, mixed hyperlipidemia, hyperchylomicronemia, or other and unspecified hyperlipidemia (ICD-9: 272.1x, 272.2x, 272.3x, 272.4x, • ICD -10:E78) | Triglycerides (TG) ≥150 mg/dL (1.7 mmol/L) |

Supplementary Table 2

| Risk Factor | Medication |
| --- | --- |
| Elevated fasting glucose | Insulin - Aspart, Lispro, Glargine, Detemir Sulfonylureas - Tolbutamide, Glipizide, Glyburide, Glimepiride Glinides - Repaglinide, Nateglinide Metformin Thizaolidinediones - Pioglitazone, Rosiglitazone GLP-1 Analogs - Exenatide, Liraglutide, Dulaglutide, Albiglutide DPP-4 inhibitors - Alogliptin, Sitagliptin, Saxagliptin, Linagliptin SGLT2 inhibitors - Canagliflozin, Dapagliflozin, Empagliflozin Alpha Glucosidase inhibitors - Acarbose Miglitol  Meglitinides - Repaglinide, Nateglinide  Amylin - Pramlintide |
| Elevated blood pressure | Aldosterone Antagonists - Eplerenone, Spironolactone Alpha Blockers - Doxazosin, Prazosin, Terazosin, Clonidine, Methyldopa ACE Inhibitors - Captopril, Enalapril, Fosinopril, Lisinopril, Perindopril, Quinapril, Ramipril, Trandolapril Angiotensin Receptor Blockers (ARBs) - Losartan, Telmisartan, Valsartan, Irbesartan, Candesartan, Olmesartan, Azilsartan Beta Blockers - Atenolol, Bisoprolol, Carvedilol, Labetalol, Metoprolol, Propranolol, Timolol, Nebivolol, Sotalol Calcium Channel Blockers- Amlodipine, Felodipine, Nicardipine, Nifedipine, Diltiazem, Verapamil, Isradipine, Lacidipine Loop Diuretics- Bumetanide, Furosemide, Torsemide, Ethacrynic Acid Thiazide Diuretics- Chlorthalidone, Hydrochlorothiazide, Indapamide, Metolazone Potassium-Sparing Diuretics - Amiloride, Triamterene Renin Inhibitors – Aliskiren |
| Elevated triglycerides and Low HDL-C | Statins - Atorvastatin, Simvastatin, Rosuvastatin, Pravastatin , Lovastatin, Fluvastatin, Pitavastatin  BA Acid Seq - Cholestyramine, Colesevelam, Colestipol  Fibrates – Gemfibrozil, Fenofibrate, Fenofibric acid Niacin PCSK9 Inhibitors - Alirocumab, Evolocumab Omega-3 Fatty Acid Supplements |
